# Physical Binding of Endothelial MCAM and Neural Transmembrane Protease Matriptase—Novel Cell Adhesion in Neural Stem cell Vascular Niche

**DOI:** 10.1038/s41598-017-05131-4

**Published:** 2017-07-10

**Authors:** Hsiu-Hui Tung, Sheau-Ling Lee

**Affiliations:** Institute of Cellular and Systems Medicine, National Health Research Institutes, 35 Keyan Road, Zhunan Town, Miaoli County, 35053 Taiwan, R.O.C.

## Abstract

Brain neural stem cells and transit amplifying cells in the subventricular zone (SVZ) of the lateral ventricles are in direct contact with the microvascular endothelium. The mechanisms/molecules of direct cell contact in the SVZ neurovascular niche are not fully understood. We previously showed that neural stem/progenitor (NS/P) cells induce brain endothelial signaling in direct cell contact through matriptase (MTP) on NS/P cell surface. In the present study, using pull-down and LC-MS/MS, we identified melanoma cell adhesion molecule (MCAM) the brain endothelial molecule that interacts with MTP. MCAM physically binds to the CUB domains of MTP and induces a chain of brain endothelial signaling including p38MAPK activation, GSK3β inactivation and subsequently β-catenin activation; none of these signaling events occurred when either MTP or MCAM is deleted. MTP-MCAM binding and induction of endothelial signaling were all sensitive to cholera toxin. Together, we identified key molecules that may represent a mechanism in neural stem cell vascular niche regulation.

## Introduction

Mammalian brain neural stem cells reside in the subventricular zone (SVZ) of the lateral ventricle (LV) within niches that consist of a specialized vascular network^1, 2^ and multiciliated ependymal cells on the ventricular surface^3^. Endothelial secreted factors have been shown *in vitro* to exhibit regulatory effects on NS/P cell proliferation^4^. *In vivo*, neural stem cells (type B cells) and transit amplifying cells (type C cells) in the LV-SVZ are in direct contact with endothelial cells of the microvasculature at sites devoid of coverage by astrocytes and pericytes^2^. Normal neurogenesis and injury-induced regeneration occur at these neurovascular contact sites^2^. The function of neurovascular direct cell contact and its molecular mechanisms have just emerged in recent years. Direct cell-cell contact with endothelial cells can regulate NS/P cell differentiation^5, 6^. It has also been shown that direct cell-cell contact with endothelial cells suppresses the cell cycle and maintains neural stem cell quiescence^7^. Different molecular interactions at the contact sites may influence neural stem cell fates/functions in different ways. Contact communication between NS/P cells and endothelial cells is a two-way street, each cell type regulates the behavior of the other to facilitate adequate neurogenesis. We recently reported that type II transmembrane serine protease matriptase (MTP) in brain is expressed in NS/P cells^8^. It promotes NS/P cell differentiation and motility^8, 9^. Importantly, MTP plays a critical role in cell-contact signaling between NS/P and brain endothelial (bEnd) cells^6^. We showed that contact co-culture of NS/P cells and bEnd cells induces a cholera toxin (CTX)-sensitive (an inhibitor of Gs-protein system) activation of endothelial p38MAPK which leads to endothelial cytokine/chemokine including IL6, IL24 and CXCL10 expression and secretion^6^. All of these cell contact-induced brain endothelial responses critically depend on the presence of MTP in NS/P cells. Some of the cell contact-induced endothelial cytokines/chemokines, such as IL6, can act on NS/P cells to induce differentiation^6^. In the present study, we describe the identification of melanoma cell adhesion molecule (MCAM) to be the brain endothelial surface molecule that interacts with neural MTP. We reveal that these two surface molecules, each on NS/P cells and bEnd cells, physically bind to each other to induce a chain of endothelial signaling from a CTX-sensitive system to endothelial p38MAPK activation, GSK3β inactivation and subsequent β-catenin activation. This molecular system represents a key mechanism of reciprocal cell-cell contact signaling between NS/P cells and bEnd cells.

## Results

### NS/P cell surface MTP induces activation of bEnd cell signaling

**Figure 1:**
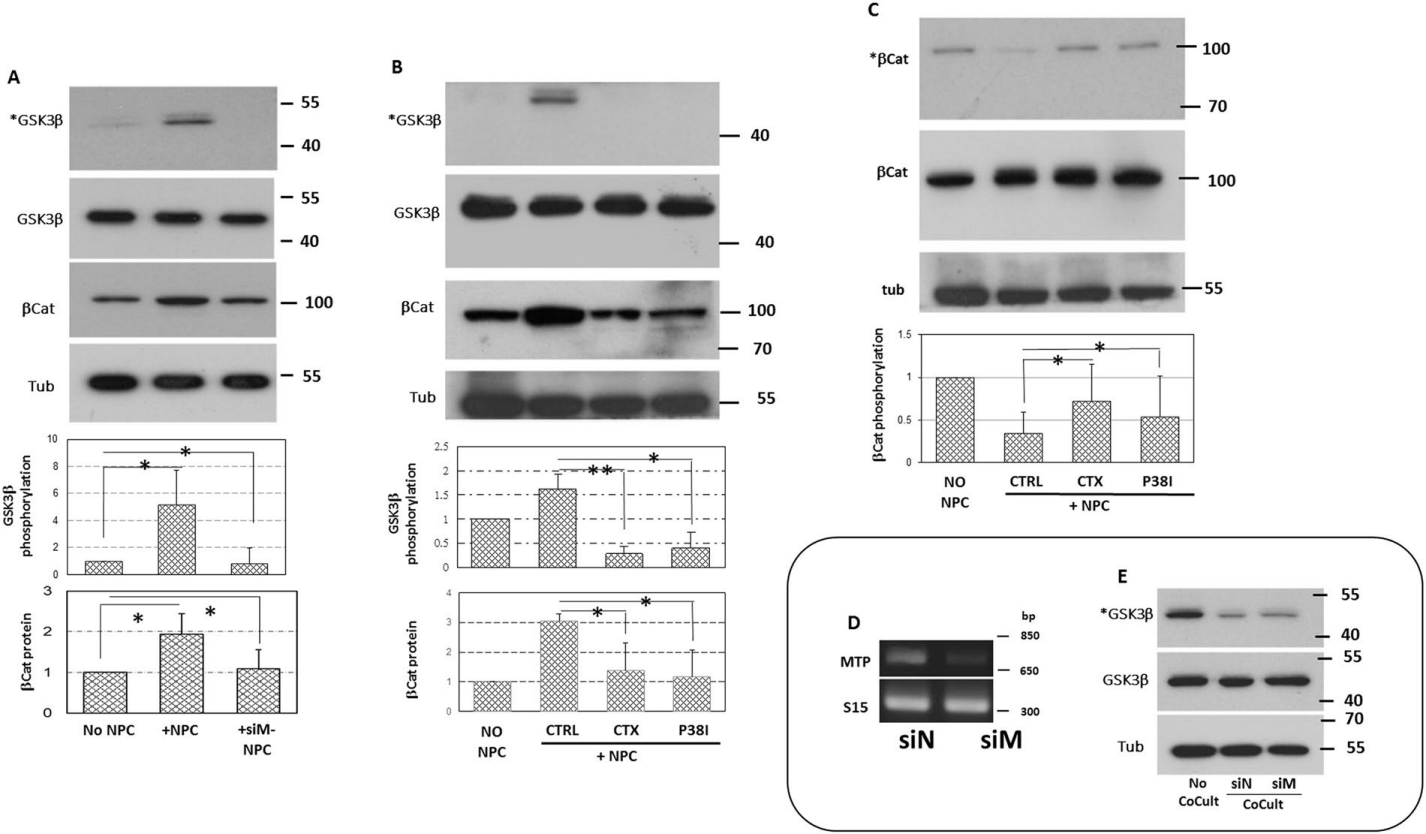
MTP-dependent endothelial GSK3β inactivation-β catenin activation is downstream of p38MAPK activation and sensitive to CTX. (**A**) Western blot of endothelial GSK3β and β-catenin. The bEnd cells were either cultured alone (No NPC), in contact co-culture with NS/P cells (+NPC) or in contact co-culture with MTP-knockdown NS/P cells (+siM-NPC). Total GSK3β protein (GSK3β), the serine 9 phosphorylated GSK3β protein (*GSK3β) and total β-catenin protein (βCat) were examined in bEnd cells. (**B**) Western of total GSK3β protein (GSK3β), the phosphorylated GSK3β protein (*GSK3β) or total β-catenin protein (βCat) in bEnd cells either cultured alone (NO NPC) or in contact co-culture with NS/P cells (+NPC) in the absence (CTRL) or in the presence of CTX (CTX) or p38MAPK inhibitor (P38I). (**C**) Western of phosphorylated and total β-Catenin (*βCat, βCat respectively) in bEnd cells cultured under the same conditions as in “B”. Bar graph showing Western blot densitometry data collected from 3–5 independent experiments. The GSK3β phosphorylation was normalized to total GSK3β. Expression level of β-catenin total protein was normalized to α-tubulin (Tub). In panel C, β-catenin phosphorylation level was normalized to total β-catenin protein. All were compared to cells cultured without NS/P cells which were set to 1. (**D)** RT-PCR of MTP in NS/P cells transfected with either control siRNA (siN) or MTP-siRNA (siM). (**E**) Western blot of GSK3β protein (GSK3β) or the phosphorylated GSK3β protein (*GSK3β) in NS/P cells. NS/P cells were collected from the experiments in “A”. The molecular sizes of protein markers in kDa were labeled on the right edge of each Western blots.

To identify brain endothelial surface molecules interacting with neuronal MTP, we first determined the endothelial signaling pathways that are activated depending on interaction with MTP. These information could serve as guide to the prediction of possible cell surface receivers. We used a Western blot-based screening (micro-Western) to search signaling molecules that are activated in brain endothelial cells only after contact co-culture with NS/P cells and that their activation depend on the presence of MTP in NS/P cells. Molecules obtained from this preliminary screening were further verified in regular Western blot. From antibodies covering total 144 signaling molecules, eight molecules were selected from the preliminary screening for further examination by regular Western blot. We found that only endothelial GSK3β serine residue 9 phosphorylation and β-catenin stability are induced by NS/P-bEnd cell contact and that both depend on neural MTP. As shown in Fig. 1, GSK3β serine 9 phosphorylation and β-catenin protein are higher in bEnd cells in direct cell-contact co-culture with NS/P cells (Fig. 1A, +NPC; Fig. 1B, +CTRL NPC) than that in bEnd cells cultured without NS/P cells (Fig. 1A and B, No NPC). GSK3β in NS/P cells, on the other hand, was at the phosphorylate states (Fig. 1E, NO CoCult). Phosphorylation was reduced after in contact co-culture with bEnd cells whether or not MTP was present (Fig. 1E) showing GSK3β serine 9 phosphorylation in NS/P cells, unlike that in bEnd cells, is not influenced by MTP. Knockdown of MTP in NS/P cells prevented their effects on endothelial GSK3β serine 9 phosphorylation and β-catenin protein (Fig. 1A, +siM-NPC) showing that both endothelial events depend on neural MTP in direct cell contact. GSK3β serine 9 phosphorylation is known to render the kinase inactive leading to non-phophorylation of its substrate β-catenin and thus prevents β-catenin protein degradation by proteosome. We tested if changes of β-catenin protein in bEnd cell in contact co-culture with NS/P cells are caused by changes of β-catenin phosphorylation and proteasome accessibility. Addition of proteasome inhibitor MG132 to the culture maintained β-catenin protein in bEnd cells cultured alone (Fig. 1C, βCat in NO-NPC) to the similar level as that in bEnd cells in contact co-culture with NS/P cells (Fig. 1C, βCat in +CTRL NPC). Maintenance of β-catenin protein in these cells is accompanied by reduced β-catenin phosphorylation (Fig. 1C, *βCat comparing NO NPC and +NPC CTRL). These results demonstrated that in contact co-culture with bEnd cells, NS/P cell MTP induced endothelial GSK3β inactivation, resulted endothelial β-catenin dephosphorylation and β-catenin protein stabilization. Because NS/P cell surface MTP induces CTX-sensitive p38MAPK phosphorylation in bEnd cells^6^, we investigated how these events are related by including CTX and p38MAPK inhibitor SB20358 in the culture. Induction of endothelial GSK3β phosphorylation, β-catenin phosphorylation and β-catenin protein stabilization by NS/P cells were all inhibited by CTX and by p38MAPK inhibitor (Fig. 1B and C, CTX and P38I respectively) demonstrating that GSK3β and β-catenin events are downstream signaling events of CTX-sensitive system and p38MAPK. Together, we showed that in cell contact, NS/P cells induce a chain of endothelial signaling events from CTX-sensitive system, p38MAPK activation to GSK3β inactivation leading to dephosphorylation and stabilization of β-catenin. All of these NS/P cell induced endothelial events were diminished when MTP was knocked down in NS/P cells. GSK3β/β-catenin signaling is activated by various types of cell surface signaling including that of growth factor and G-protein coupled receptors. The present data, consistent with our early studies^6^, point to the existence of a G protein system on the bEnd cell surface which receives NS/P cell signaling through interaction with MTP.

### CUB motifs of MTP are the key elements that induce brain endothelial responses in contact co-culture

**Figure 2:**
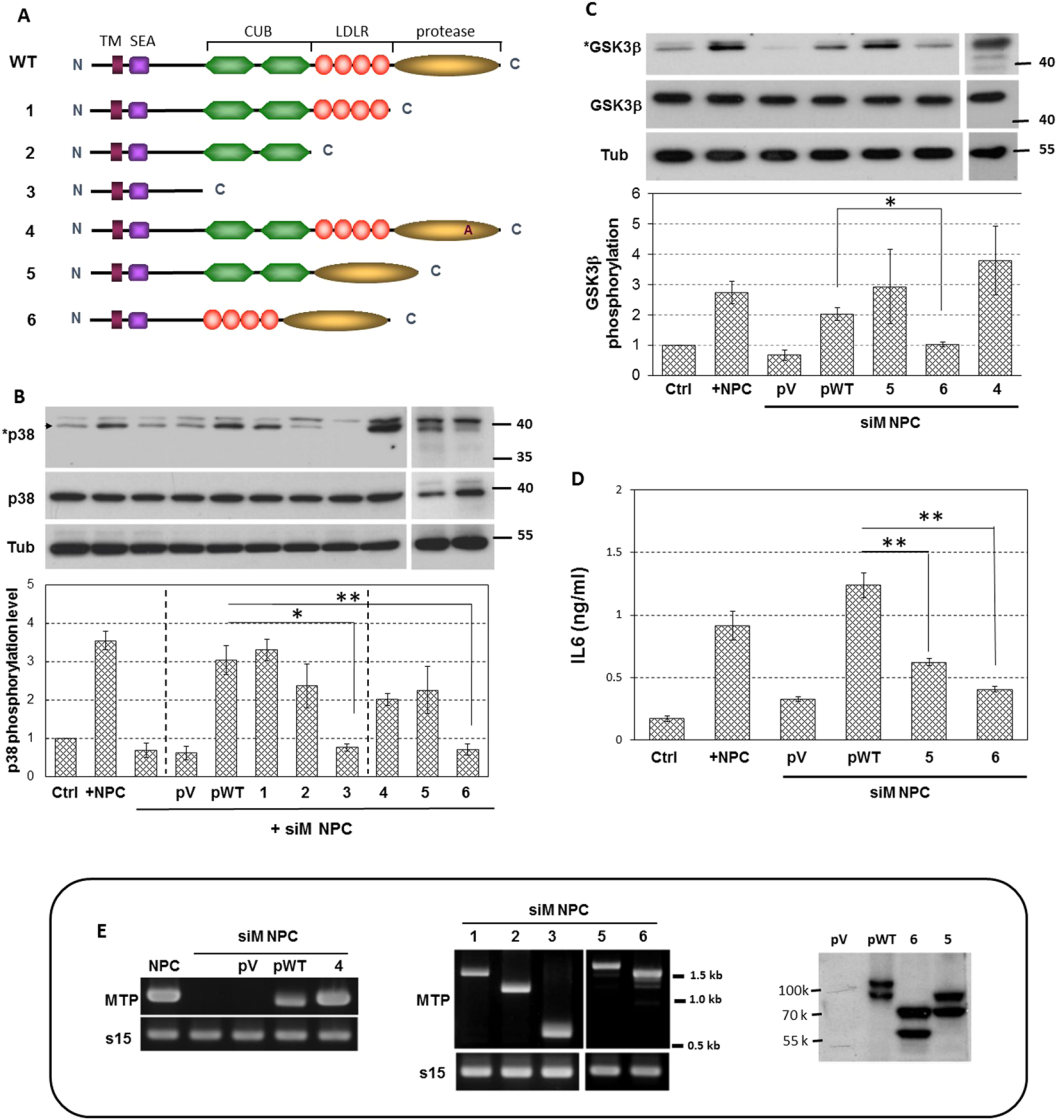
CUB motifs of MTP are the key elements for induction of endothelial signaling activation in NS/P cell and bEnd cell contact co-culture. (**A**) Schematic illustration of full-length MTP protein structure (WT) and the various deletion constructs (1–5). Variant 6 contains a point mutation in the protease domain which diminishes the protease activity. (**B**) Western blot of phosphorylated or total p38MAPK (*p38 and p38 respectively) in bEnd cells cultured alone (Ctrl), in contact co-cultured with wild type NS/P cells (+NPC), in contact co-cultured with MTP-knockdown NS/P cells (+siM NPC), or in contact co-cultured with MTP-knockdown NS/P cells carrying control empty expression vector (pV), the full-length wild-type MTP (pWT), or the various MTP constructs (1, 2, 3, 4, 5, or 6). Tub is the α-tubulin used as loading control for Western. Bar graph shows the quantitative data of the Western blot. Variants 5 and 6 were run on different gel from the others. The original blot of these samples was shown in Supplementary Fig. 1A. (**C**) Western blot of total and phosphorylated GSK3β (GSK3β and *GSK3β, respectively) in bEnd cells cultured alone (Ctrl), in contact co-cultured with wild type NS/P cells (+NPC), in contact co-cultured with MTP-knockdown NS/P cells (+siM NPC), or in contact co-cultured with MTP-knockdown NS/P cells carrying control empty expression vector (pV), the full-length wild-type MTP (pWT), or the various MTP constructs (4, 5, or 6). Variant 4 was run on different gel from the others. The original blot of this sample was shown in Supplementary Fig. 1B. (**D**) ELISA assay of IL6 in the medium of bEnd cells cultured alone (Ctrl), in contact co-cultured with wild type NS/P cells (+NPC), in contact co-cultured with MTP-knockdown NS/P cells (+siM NPC), or in contact co-cultured with MTP-knockdown NS/P cells carrying control empty expression vector (pV), the full-length wild-type MTP (pWT), or the various MTP constructs (5 or 6). (**E**) Expression of the various MTP constructs in NS/P cells used in the co-culture. Left and middle panels are RT-PCR. Images were grouped together according to the expected product size. The deletion variants have different size were thus grouped to form the middle image. Expression of the full-length MTP, variants 5 and 6 was also shown by Western in the right panel. The bar graph in B and C show densitometry data of Western blots collected from 3–5 independent experiments and analyzed as described in Fig. 1. *P value ≦ 0.1; **P value ≦ 0.05.

We further investigated the possible protein motif(s) of MTP for suspected roles of endothelial signaling responses during NS/P and bEnd cell contact. The responsive motifs were then used to isolate likely interacting-molecules from bEnd cells. The full-length MTP protein, from N-terminus to C-terminus, contains the transmembrane motif (TM), SEA (sea-urchin sperm protein-enteropeptidase-agrin) motif, two tandem repeats of CUB (Cls/Clr urchin embryonic growth factor, bone morphogenic protein-1) domains, four tandem repeats of LDLR (low-density lipoprotein receptor) domains and the serine protease domain (Fig. 2A, WT)^10^. Being a type II transmembrane serine protease, MTP sits on the cell surface with SEA, CUB, LDLR and serine protease motifs exposed to the extracellular space. We made various deletion constructs by removing different MTP extracellular domains (Fig. 2A, variants 1,2,3,5 and 6). We also made the protease inactive MTP by changing the serine residue 805 in the catalytic triad of the protease domain to alanine^9^ (Fig. 2A, variant 4). Each construct was transfected into MTP-knockdown NS/P cells separately; cells were then co-cultured with bEnd cells under cell-contact culture conditions. By examining endothelial p38MAPK phosphorylation, GSK3β serine 9 phosphorylation and IL6 secretion, we asked which of the different MTP constructs failed to restore the knockdown cells’ ability to induce bEnd cell signaling responses. Expression of these various constructs was shown in Fig. 2E. As expected, ectopic expression of the full-length MTP (Fig. 2B, C and D, pWT) but not the empty vector (Fig. 2B, C and D, pV) in MTP-knockdown NS/P cells (Fig. 2B, C and D, +siM NPC) restored their ability to induce endothelial p38MAPK phosphorylation (Fig. 2B), GSK3β serine 9 phosphorylation (Fig. 2C) and IL6 secretion (Fig. 2D). Deletion of the entire protease domain (Fig. 2A, variant 1) or the entire region beyond the CUB domain (Fig. 2A, variant 2) maintained induction of endothelial p38MAPK phosphorylation (Fig 2B, 1 and 2), but further removal of the CUB domains (Fig. 2A, variant 3) diminished this capacity (Fig. 2B, lane 3). These results suggest that the protease function or the LDLR domains are not required, but the CUB domains may be essential for NS/P cells to influence endothelial responses. Indeed, ectopic expression of the protease inactive full-length MTP (Fig. 2A, variant 4) or MTP lacking the four LDLR domains (Fig. 2A, variant 5) restored the MTP-knockdown NS/P cells’ ability to induce endothelial p38 MAPK phosphorylation (Fig 2B, 4 and 5) and GSK3β serine 9 phosphorylation (Fig 2C, 4 and 5), whilst MTP lacking only the two CUB domains failed to do so (Fig. 2A, variant 6; 6 in Fig 2B,C and D). All together, these data demonstrate that CUB motifs of MTP are the key elements for NS/P cell interaction with bEnd cells and induction of endothelial cell signaling. Unexpectedly, although the lack of LDLR domains in MTP did not impair its ability to induce endothelial p38MAPK phosphorylation (Fig. 2B, lane 5), it did cause the loss of its ability to induce endothelial IL6 expression (Fig 2D, 5). It seems, LDLR domains may be involved in IL6 induction through some p38MAPK-independent mechanism.

### MTP CUB motifs isolate endothelial melanoma cell adhesion molecule

**Figure 3:**
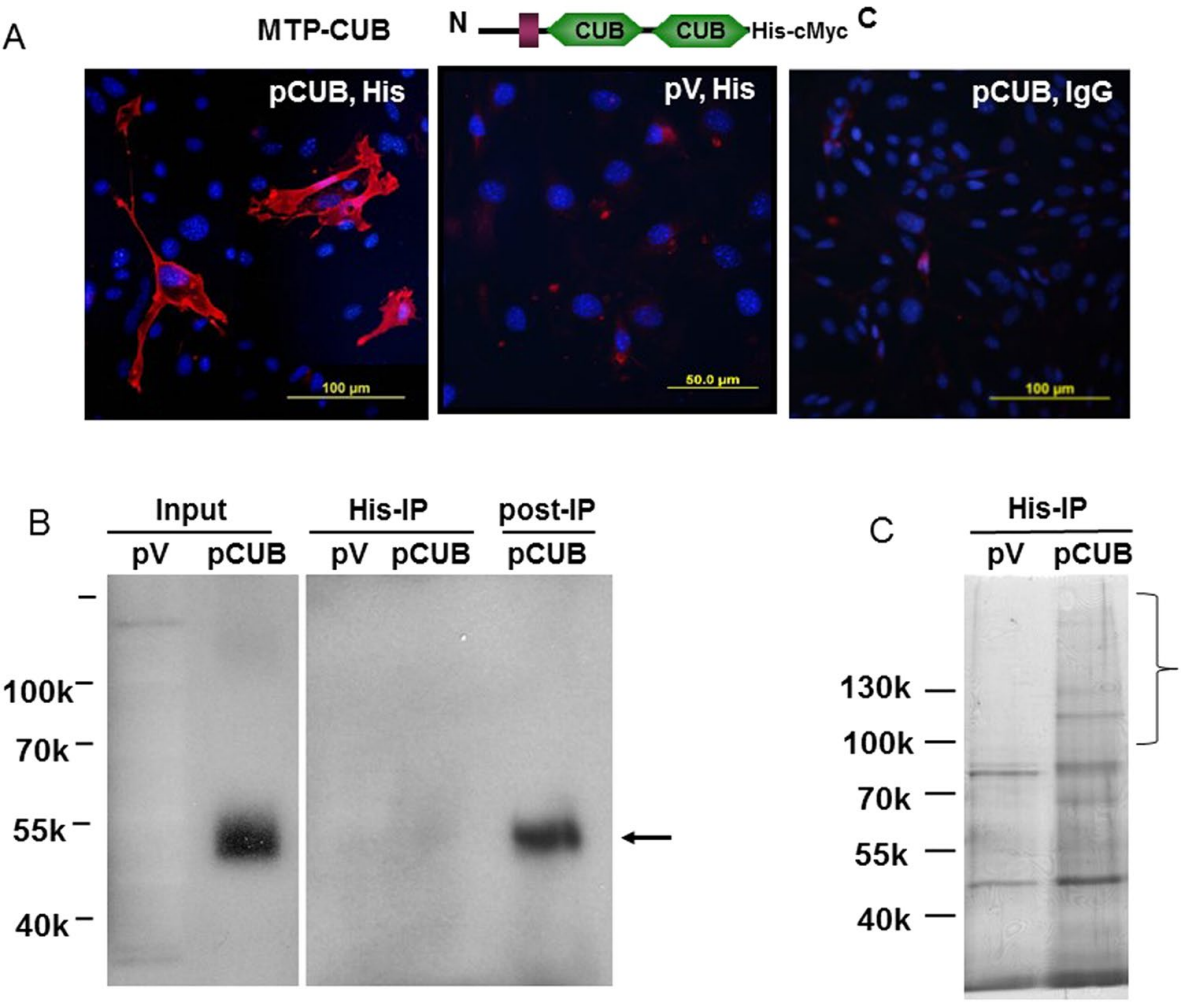
Expression of MTP CUB domains in brain endothelial cells isolates associated molecules. (**A**) Cell staining of MTP-CUB. Brain endothelial cells transfected with plasmid expressing MTP-CUB domains (structure shown in the diagram) carrying 6His and c-Myc tags (pCUB) or with the same expression vector without insert (pV). Cells were immunostained, without Triton X-100 treatment, with anti-His antibody (His) or control IgG of the same subtype (IgG). Nuclei are shown in blue. (**B**) Western blot detection of MTP-CUB in lysates prepared for “A” using anti-Myc antibody. Input: the total lysate of cells carrying MTP-CUB expression vector (pCUB) or the empty vector (pV). His-IP: anti-His antibody immunoprecipitated cell lysates released by DTT treatment. After releasing MTP-CUB associated protein (His-IP of pCUB), MTP-CUB protein was released from the antibody beads by urea in acidic condition that is shown in the lane labeled “post-IP pCUB”. Arrow indicates the expected CUB protein. (**C**) Silver staining of the proteins pulled down by anti-His.

Data presented in the previous section clearly demonstrates that NS/P cells induced endothelial responses are mediated by the CUB domains of MTP. bEnd cells express little or no MTP^6^. Induction of endothelial p38MAPK phosphorylation and cytokine/chemokine production by NS/P cells can be replicated by ectopic expression of MTP in bEnd cells^6^ suggesting ectopic MTP interacts with its receiver molecules in a similar way as that in NS/P-bEnd cell contact. We reasoned that expression of MTP CUB domains in bEnd cells is a feasible way to pull down MTP-interacting endothelial molecules that can be identified by liquid chromatography (LC) with mass spectrometry (MS) analysis. The bEnd cells were transfected with an MTP variant containing the transmembrane domain and the two CUB domains but no other extracellular motifs (MTP-CUB, diagram illustration in Fig. 3). This construct expressed MTP-CUB protein carrying both 6His and c-Myc tag proteins at the C-terminus which allow detection by antibody against either tag protein. Cells transfected with this construct expressed MTP-CUB protein both in the cytosol and on the cell surface judged by cell staining (Tung and Lee unpublished observation). Cell membrane localization of MTP-CUB protein was further confirmed by immunostaining of the fixed cells without Triton X-100 treatment which permits only the proteins exposed at the cell surface to be stained with antibody. Figure 3A showed the results of immunostaining with antibody to the tag protein (His) where only cells transfected with vector carrying MTP-CUB (pCUB) show positive staining. Neither the cells expressing MTP-CUB immunostained with a control IgG of the same subtype as tag protein antibody (Fig. 3A, pCUB, IgG) nor the cells transfected with the control empty vector immunostained with the tag antibody (Fig. 3A, pV, His) showed any positive staining. Western blot detected MTP-CUB protein of the expected molecular mass in cells transfected with vector carrying MTP-CUB (Fig. 3B, Input pCUB), but not in cells transfected with the empty vector (Fig. 3B, Input, pV). Both immunostaining and Western blot confirmed the specific expression of MTP-CUB in the transfected cells. Considering that MTP-CUB targeted proteins may either directly bind to CUB, indirectly bind to CUB through a third party or transiently interact with CUB, cells expressing MTP-CUB were treated with dithiobissuccinimidyl propionate (DSP) to cross-link all proteins that are in physical proximity with MTP-CUB. After IP MTP-CUB with anti-His antibody that had been linked to the protein-G beads by dimethyl dihydrochloride, MTP-CUB associated molecules can be dissociated with dithiothreitol (DTT) without disturbing antibody-bound MTP-CUB. This approach can also avoid high amount of MTP-CUB in the pull-down to affect LC-MS/MS readout.

DTT treatment of the immunocomplex from cells carrying MTP-CUB revealed multiple protein bands on silver stained gel (Fig. 3C pCUB) that were not found from cells transfected with control vector (Fig. 3C pV). Western blot by antibody to the tag protein c-Myc of DTT released materials showed no protein band in cells transfected with either the empty vector (Fig. 3B, His-IP, pV) or with MTP-CUB construct (Fig. 3B, His-IP, pCUB). Whilst MTP-CUB protein was largely retained on binding to the antibody-protein G beads and was only released by urea treatment under acidic conditions (Fig. 3B, post-IP, pCUB). Clearly, DTT treatment released CUB-associated proteins with minimal amount of CUB protein which may otherwise interfere with LC-MS/MS readout. The molecular mass of most MTP-CUB associated proteins was larger than 100 kDa (Fig. 3C, pCUB). LC-MS/MS peptide score distribution showed that there is 163 peptide matches above identity threshold (p ≦ 0.05) from 2636 quires. Peptides matched to 57 known proteins. Some of these were high abundant house-keeping proteins. There were, however, no known G-protein coupled receptors or any G-protein related proteins. Since our data so far have pointed to the presence of endothelial cell surface receiver for neural MTP, we were particularly interested in molecules that may function as signaling receptors. Membrane-associated or transmembrane molecules had LC-MS/MS score higher than 91. These molecules were thus selected for further examination.

### Endothelial MCAM and neural MTP are critical for bEnd and NS/P cell contact interaction

**Figure 4:**
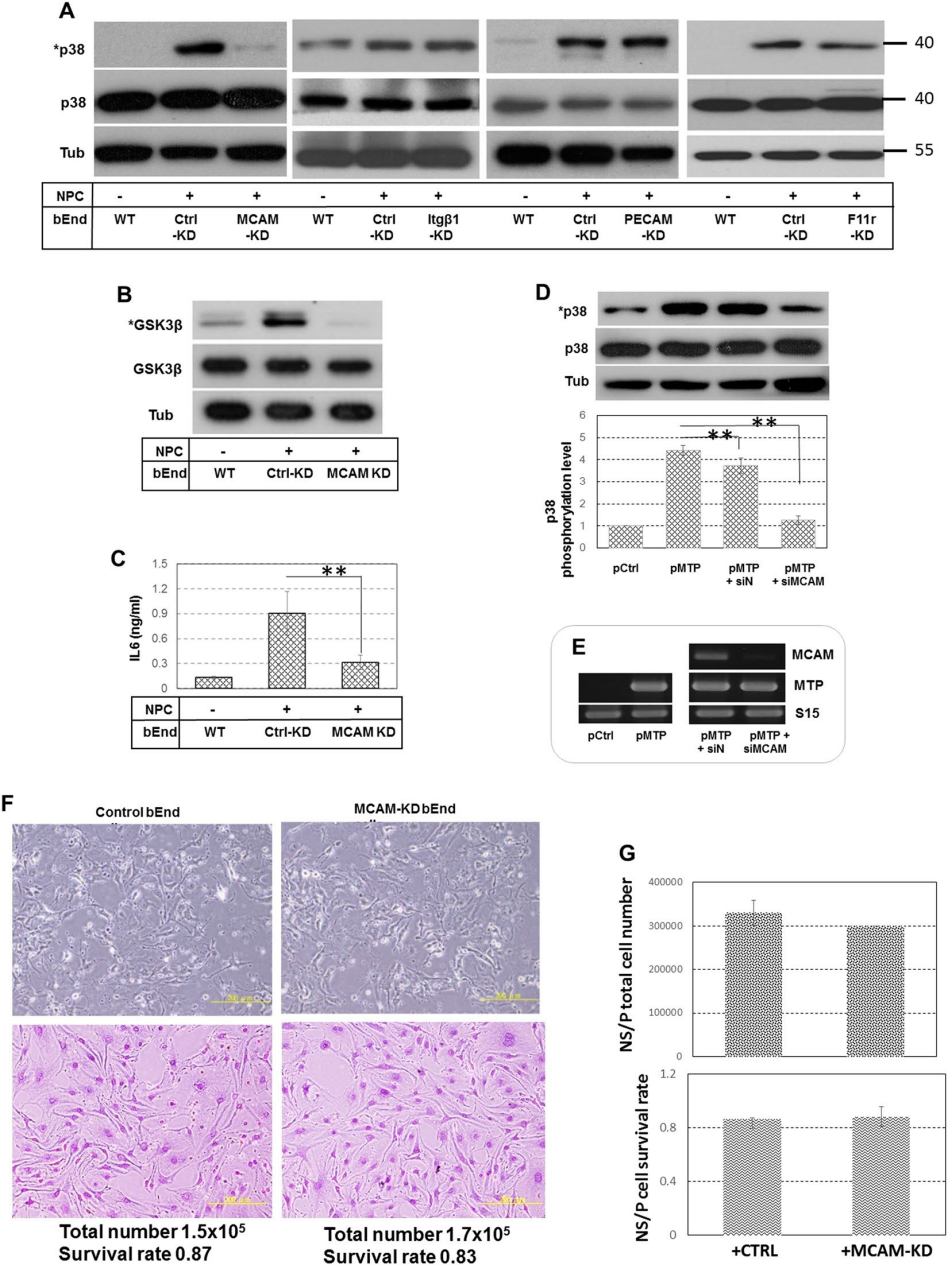
Presence of both MTP and MCAM are essential for NS/P cells and bEnd cells contact interaction. (**A**) Western blot of endothelial p38MAPK. Total p38MAPK (p38) and phosphorylated p38MAPK (*p38) were examined in wild-type bEnd cells (WT), control siRNA transfected bEnd cells (Ctrl-KD), MCAM knockdown bEnd cells (MCAM KD), integrin β1 knockdown bEnd cells (itgβ1-KD), PECAM knockdown bEnd cells (PECAM-KD) or F11r knockdown bEnd cells (F11r-KD) cultured alone or in contact co-culture with NS/P cells (− or +NPC, respectively). The same results were observed in three independent experiments. (**B**) Western blot of endothelial GSK3β. GSK3β serine 9 phosphorylation (*GSK3β) and total GSK3β protein in wild-type (WT), control siRNA transfected (Ctrl-KD) or MCAM knockdown (MCAM KD) bEnd cells cultured alone or in contact co-culture with NS/P cells (− or +NPC, respectively). (**C**) ELISA of endothelial IL6 in the medium of wild-type (WT), control siRNA transfected (Ctrl-KD) or MCAM knockdown (MCAM KD) bEnd cells cultured alone or in contact co-culture with NS/P cells (− or +NPC, respectively). Data were collected from three independent experiments. (**D**) Western of p38MAPK in bEnd cells transfected with an empty expression plasmid (pCtrl), with the MTP expression plasmid (pMTP), with the MTP expression plasmid plus a control siRNA (pM + siN), or with the MTP expression plasmid plus MCAM-specific siRNA (pM + siMCAM). (**E**) RT-PCR of MCAM and MTP from cells used in the experiments in “D”. (**F**) Representative images of bEnd cells. Cell images on the top are live cells taken 48 hours after transfected with control siRNA (Control bEnd) or MCAM-siRNA (MCAM-KD bEnd) and before they were proceeded to co-culture. Cell images at the bottom were taken 24 hours after co-culture with NS/P cells. NS/P cells were removed, bEnd cells were fixed and stained with crystal violet. Scale bars in the images represent 200 um. The total bEnd cell number and cell survival rate were listed at the bottom of the images. These are average number of 2–3 independent assays. **G**. Total NS/P cell number (graph on the top) and cell survival (graph at the bottom) after co-culture with bEnd cells transfected with control siRNA (+CTRL) or MCAM-siRNA (+MCAM-KD).

Membrane associated molecules including melanoma cell adhesion molecules (MCAM), integrin β1 plate endothelial cell adhesion molecule (PECAM) and F11 receptor (F11r, junctional adhesion molecule A) selected from LC-MS/MS were examined in detail for their interaction with MTP in NS/P cell and bEnd cell contact co-culture. The scores and the number of matched peptides of these molecules were listed in Supplementary Table 1. These molecules were knocked down individually in bEnd cells; cells were co-cultured in contact with NS/P cells; the endothelial responses described in previous sections were examined to determine which protein(s) is (are) required for NS/P cell-bEnd cell interaction. Knock down of either integrin β1, PECAM or F11r did not affect NS/P cell contact induction of endothelial p38MAPK activation (Fig. 4A). These investigations revealed that MCAM exhibited significant effects on MTP-mediated NS/P-bEnd cell contact interaction. When MCAM was knocked down in bEnd cells, neither bEnd cell p38MAPK phosphorylation (Fig. 4A, MCAM-KD), GSK3β phosphorylation (Fig. 4B, MCAM-KD), nor IL6 production (Fig. 4C, MCAM-KD) was induced by contact with NS/P cells. These are exactly the same results when MTP was knocked down in NS/P cells (Fig. 2B and D, also see data in ref. 6) which supports the interaction between neural MTP and endothelial MCAM. MTP ectopically expressed in bEnd cells binds to MCAM (previous section) and replicates MTP-dependent cell-contact responses^6^ suggesting similar molecular interaction in these cells as that between NS/P cell MTP and endothelial MCAM. Experiments combining ectopic expression of MTP and MCAM knockdown in bEnd cells were therefore carried out to further demonstrate that MTP and MCAM are both required to be present to induce endothelial signaling. Brain endothelial bEnd cells normally do not express MTP (Fig. 4E, pCtrl), thus they have low p38MAPK phosphorylation (Fig. 4D, pCtrl, also shown in Fig. 4A WT and in ref. 6). Ectopic expression of full-length MTP (Fig. 4D, pMTP) in bEnd cells, but not the empty control vector (Fig. 4D, pCtrl) induced p38MAPK phosphorylation. Importantly, knockdown of MCAM in bEnd cells completely reversed ectopic MTP induced effect (Fig. 4D, pMTP +siMCAM). Evidently, collaborative interaction between NS/P cell MTP and bEnd cell MCAM is a critical factor guiding communication between the two types of cells under cell contact culture conditions; loss of either molecule resulted in loss of communication. There was no obvious change of cell morphology in MCAM-knockdown bEnd cells (Fig. 4F two images on the top, compare MCAM-KD bEnd with control bEnd). After co-culture with NS/P cells in contact condition, MCAM-knockdown bEnd cells remained similar morphological feature as the control bEnd cells (Fig. 4F, two images at the bottom). Cell adhesion between bEnd cells or adhesion to the culture surface also appeared not to be changed much by MCAM-knockdown (Fig. 4F). Total cell number and the cell survival rate of control and of MCAM-knockdown bEnd cells after co-culture with NS/P cells were roughly the same (Fig. 4F). Although siRNA transfection may not knock down MCAM expression completely in every cell, these observations do imply that MCAM has no significant effect on bEnd cell morphology, self-adhesion or growth. NS/P cell number (Fig. 4G, graph on the top) or NS/P cell survival rate (Fig. 4G, graph at the bottom) were not much different after contact co-culture with control (Fig. 4G, +CTRL) or with MCAM-knockdown (Fig. 4G, +MCAM-KD) bEnd cells. Together, all results showed that MCAM and MTP interaction has little influence on cell growth of either cell type but mainly maintains both cells in contact position.

**Figure 5:**
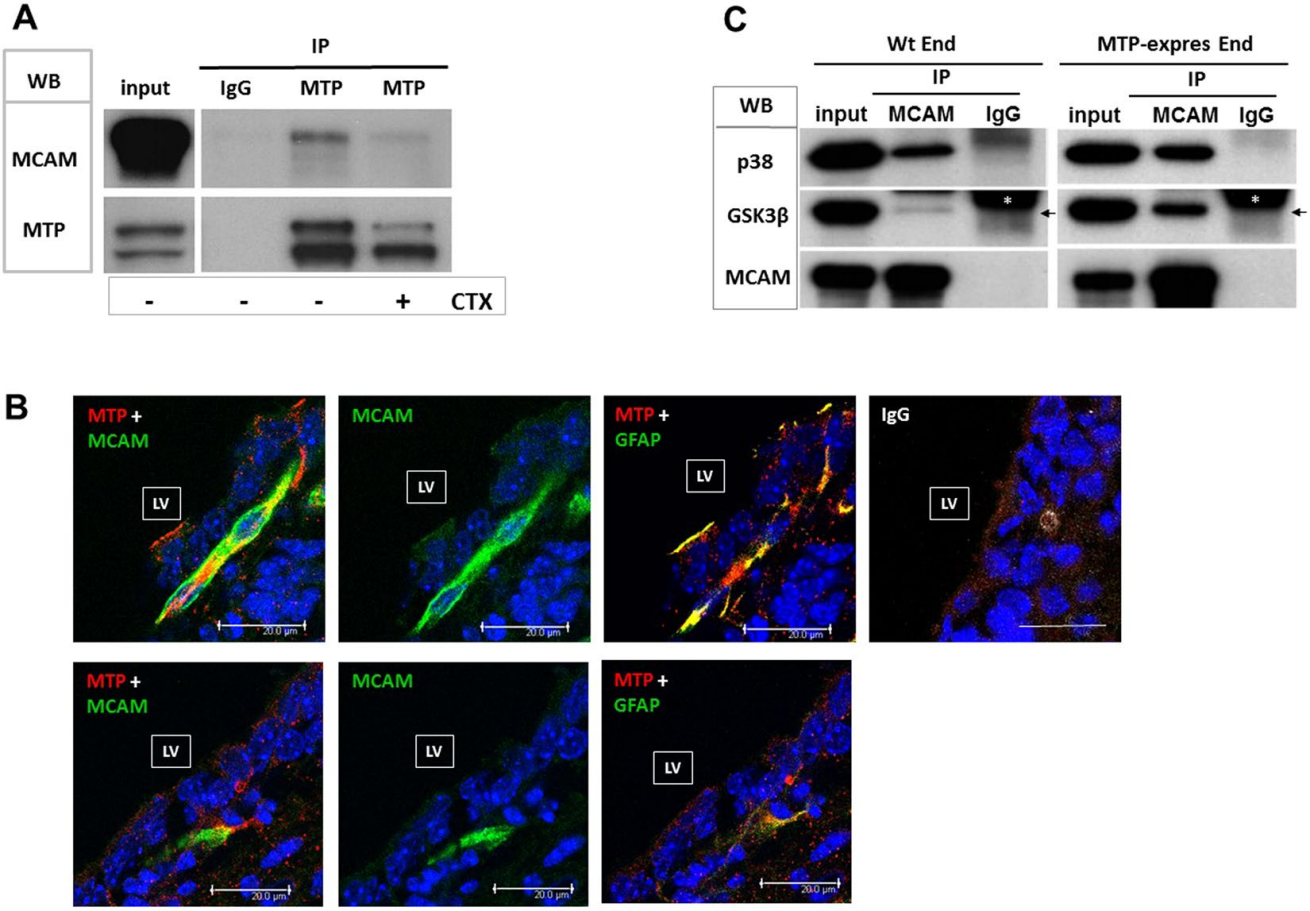
MTP and MCAM physically bind to each other and the binding is impaired by CTX. (**A**) MTP IP-MCAM Western of bEnd cells transfected with full-length MTP carrying both 6His and c-Myc tag proteins. Cell lysate were IP with c-Myc antibody to pull down MTP (MTP IP) or with control IgG of the same subtype (IgG IP) followed by Western blot (WB) with MCAM (MCAM) or 6His antibody (MTP). Cell culture in the absence or presence of CTX is indicated at bottom of the image (-, + CTX). input: Western blots of the MTP-expressing bEnd cell lysate before IP. This figure is cropped from the original blot that is shown in Supplementary Fig. 2. The same results were observed in 3 independent experiments. (**B**) Brain tissue staining of MTP and MCAM. Images were taken from SVZ of the mouse brain lateral ventricle. Tissues were stained simultaneously with antibodies to MTP, MCAM and GFAP from different host species and visualized by Alexa 594, Alexa 488 and Cy5-conjugated secondary antibodies, respectively. GFAP is used to label SVZ NS/P cells. In GFAP and MTP combined image, GFAP staining was pseudo-colored in green for better viewing. LV indicates the location of lateral ventricle. IgG shows immunstaining with control IgG of the same subtypes. Scale bars represent 20 um. (**C**) Western blot of MCAM IP from bEnd cells (Wt-End) or bEnd cells transfected with full-length MTP carrying both 6His and c-Myc tag proteins (MTP-expres End). Cell lysates were IP with MCAM antibody (IP MCAM) or with control IgG of the same subtype (IP IgG) followed by Western blot (WB) with antibody to p38, GSK3β or MCAM. A small portion of cell lysates were subjected to WB without IP as input control (input). Arrows localize the expected protein bands. White stars indicate the location of IgG.

### MCAM and MTP physically bind to each other and the binding is cholera toxin (CTX)-sensitive

Immunoprecipitation (IP) followed by Western blot (WB) was carried out to further verify if MCAM binds directly to MTP or interacts with it through association with third parties. Since ectopic MTP expressed in bEnd cells interacts with endothelial MCAM (previous section), vector expressing full-length MTP carrying both 6His and cMyc tag proteins at the C-terminus was transfected into bEnd cells. IP of MTP using antibody to cMyc tag protein (Fig. 5A, IP MTP), but not using control IgG (Fig. 5A, IP IgG), pulled down MCAM (Fig. 5A, WB MCAM). Direct binding between MTP and MCAM was completely disrupted by CTX treatment (Fig. 5A, + CTX). Clearly, MTP and MCAM are directly bound to each other in the cells and a Gs-protein system is critical for the interaction. In mouse brain lateral ventricular SVZ, MTP is expressed in GFAP-positive type B and C NS/P cells (Fig. 5B, MTP + GFAP). Type B cells contact to ependymal cells in one end and to blood vessel at the other end (Fig. 5B images on the top panels)^1–3^. Type C cells are at the blood vessel contact sites (Fig. 5B, images at the bottom panels)^1, 2^. Both type of NS/P cells were closely in contact with MCAM-expressing endothelial cells (Fig. 5B, MTP + MCAM). P38MAPK and GSK3β phosphorylation, although, were induced by MTP-MCAM interaction (previous sections), both proteins were not found in MTP-CUB pulldown LC-MS/MS data sets suggesting both proteins were not in direct contact with MTP. Figure 5C showed that p38MAPK in bEnd cells is co-IP with MCAM (Fig. 5C, p38 in Wt-End), whilst little GSK3β is pulled down with MCAM (Fig. 5C, GSK3β in Wt End). P38MAPK was maintained binding to MCAM in cells express ectopic MTP (Fig. 5C, p38 in MTP-expres End). Interestingly, GSK3β was also co-IP with MCAM in MTP-expressing bEnd cells (Fig. 5C, GSK3β in MTP-expres End). All together, these studies showed that upon binding of MTP and MCAM at cell surface, in addition to p38MAPK, GSK3β also bind to MCAM intracellular part and is inactivation phosphorylated by p38MAPK to prevent β-catenin phosphorylation and degradation.

## Discussion

**Figure 6:**
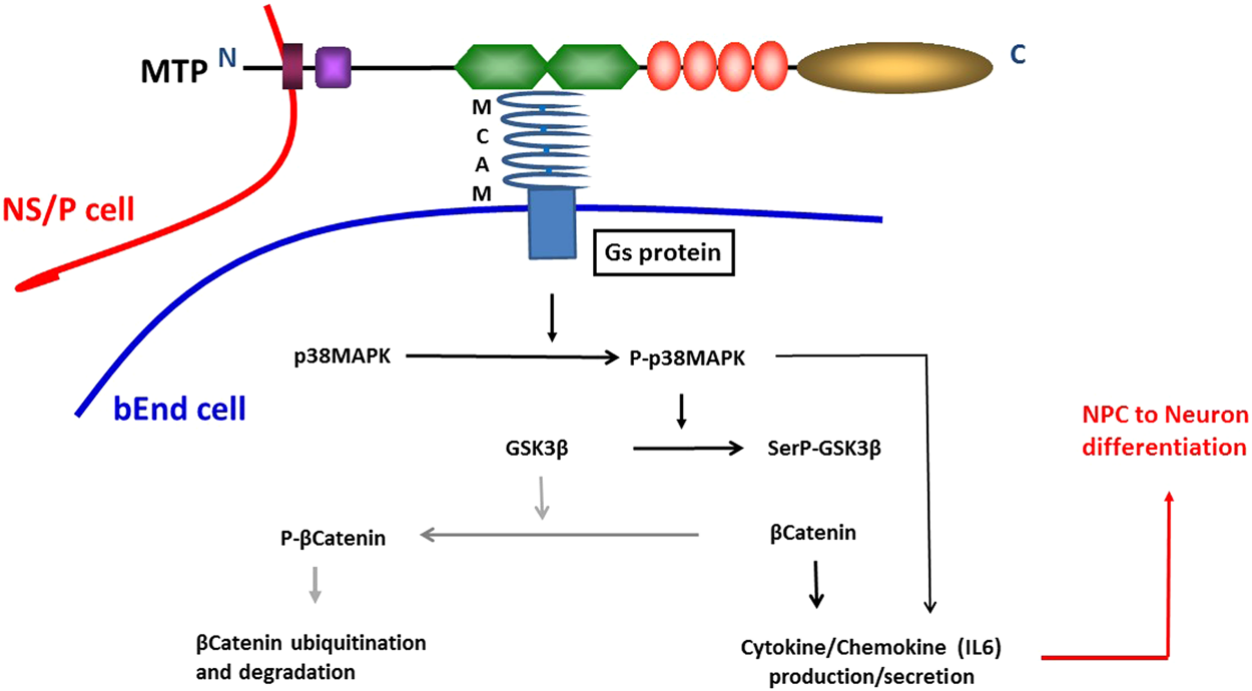
Schematic diagram of MTP and MCAM mediated NS/P cells and brain endothelial cells contact interaction. MTP-MCAM binding induced events are indicated by black arrows. Grey arrows indicate the resting state.

Cell adhesion is one key element through which stem cells communicate with their local environment. In brain SVZ, direct contact of neural stem cells and transit amplifying cells to endothelial cells is important for stem cell function^1, 2^. In this study, using the endothelial response we previously established as readout^6^, we identified MCAM-MTP interaction as a novel cell-contact molecular mechanism between brain endothelial cells and neural stem/progenitor cells for direct cell-cell communication. We determined a series of endothelial responsive signaling stimulated by NS/P cell MTP. We previously showed that NS/P cells isolated from adult mouse brain SVZ can only induce MTP-dependent endothelial signaling under cell-contact culture conditions^6^. These cells contain a large number of type C cells and relatively small number of B cells. MTP is present in both type B and C SVZ cells. These results implied that both type B and C SVZ cells contact endothelial cells through MTP-MCAM direct binding. This is supported in mouse brain tissue staining (Fig. 5D) where MTP was found in both type B and type C cells in close vicinity to MCAM-positive blood vessel. It was recently suggested that combinatory binding of neural Eph and Notch to endothelial ephrin B2 and Jagged1 is a feature of the quiescent adult SVZ B cells rather the transient amplifying C cells^7^. Binding of neural α6β1 integrin to endothelial laminin appears to behave the same^1^. Although we did not specify if physical binding between neural MTP and endothelial MCAM differentially affects type B or type C cell behavior, our studies did not exclude the possibility that MTP-MCAM binding, same as the two aforementioned molecular systems, may function to maintain the type B cell population. This question would be addressed adequately using type B and type C cells in separate co-culture studies. The finding that disruption of MCAM-MTP interaction does not increase NS/P cell proliferation in NS/P-bEnd cell co-culture (Fig. 4G) seems to favor the possibility that MTP-MCAM binding mainly functions to hold NS/P cells in contact with bEnd cells, which may facilitate stimulation of or response to vascular effectors. We have demonstrated that MTP-mediated activation of brain endothelial signaling induces a number of endothelial chemokines/cytokines possessing differentiation or proliferation activities^6^. One of such molecules, the endothelial IL6 can promote NS/P cell differentiation^6^. Our studies present a model that (Fig. 6) at the cell-contact sites, MTP on NS/P cell surface, through its extracellular CUB domains, binds to the endothelial surface MCAM. This binding initiates a CTX-sensitive Gs protein-dependent activation of p38MAPK that binds to MCAM inside bEnd cells. GSK3β is recruited to MCAM and phosphorylated at Ser9 by p38MAPK which renders GSK3β kinase inactive leaving its substrate β-catenin non-phophorylated and thus preventing β-catenin protein degradation by proteosome. Activated p38MAPK^6^ and stabilized β-catenin may both induce the expression of endothelial cytokine/chemokine including IL6. Secreted from bEnd cells, IL6 promotes adjacent NS/P cell differentiation to neuron^6^. MTP-MCAM interaction is one of the few cell-contact mechanisms between NS/P cells and brain endothelial cells^1, 7^. This molecular interaction may provide important reciprocal regulation in SVZ neural stem cell vascular niche.

G protein-coupled signaling is mainly activated by the seven-transmembrane receptors^11^, but single transmembrane spanning GPCRs including the receptor tyrosine kinases for EGF and insulin/insulin-like growth factor, the amyloid precursor protein, the C-type natriuretic peptide receptor, the zona pellucida glycoprotein receptor, the integrin and the T cell receptor are well known^12^. Although our data indicate the involvement of an endothelial G protein in MTP-mediated neural stem cell vascular niche cell contact interaction, we did not find any of the known G protein-coupled receptors in MTP isolated molecules. Interestingly, we found that the single transmembrane cell adhesion molecule MCAM behaves as a receptor of MTP to induce a CTX-sensitive Gs protein activator. MCAM, also known as MUC18 or CD146, is a membrane cell adhesion molecule of the immunoglobin superfamily^13^. It was originally identified in melanoma cells^14^ and functions in tumor growth and metastasis^13, 15^. MCAM is constitutively expressed in endothelium irrespective of anatomical sites^16, 17^ where it appears to play role in endothelial cell adhesion and angiogenesis^18–20^. Despite the fact that little is known about its ligand or signaling events, there are data to support the idea that MCAM is not just a cell adhesion molecule but can function as a receptor to deliver outside-in signaling. For instance, MCAM engagement by its specific monoclonal antibody in HUVEC cells can induce phosphorylation of the non-receptor tyrosine kinase Fyn which then triggers downstream Pyk2, p130Cas, FAK and paxillin activation^21^. In recent years, MCAM was shown to be a co-receptor for VEGFR2 and is required for VEGF-induction of AKT/p38MAPK/NFrB activation of endothelial migration and microvascular formation^22, 23^. The secreted protein netrin-1 was shown to bind to MCAM and to induce HUVEC activation and angiogenesis through VEGFR2, ERK1/2 and p38 phosphorylation^24^. Our studies identify MTP as a novel ligand for MCAM. Our studies also identify MCAM as a novel single transmembrane molecule linking to G protein-coupled activation and signaling transduction.

CUB and LDLR domains of MTP have long been thought to bind and bring substrates/partners to the proximal vicinity of MTP for various functions. Besides its cognate inhibitor^25^, however, other molecules that physically interact with MTP CUB or LDLR have not been described. Although proteins containing CUB domain(s) are involved in a wide range of biological functions including tissue repair, axon guidance^26, 27^, angiogenesis^26, 27^, inflammation^28^ and neurotransmission^29^, the mechanisms/roles of CUB domains themselves are largely unexplored. We report here that adhesion molecule MCAM is a binding protein for MTP CUB domains and determine the signaling activation induced by CUB-MCAM binding. How the individual domain of MCAM influence the binding to MTP or the signaling induced thereafter would be a subject of interesting in understanding neural stem cell vascular niche communication.

In summary, we identified that 1. Neural MTP and endothelial MCAM physical binding is a novel molecular mechanism in neurovascular direct cell contact interaction. 2. MTP-MCAM mediated direct cell contact represents a reciprocal regulatory mechanism in neural stem cell vascular niche where induction of endothelial responses act in turn to regulate NS/P cell behavior. 3. Transmembrane serine protease MTP is a novel ligand of cell adhesion molecule MCAM. 3. MCAM is a novel G protein related receptor. 4. MCAM is a direct binding molecule of CUB motifs.

## Methods and Materials

### Cell culture

Mouse brain endothelial cells bEnd.3 (from Bioscience Collection and Research Center, Hsinchu, Taiwan) were routinely maintained in DMEM supplemented with 10% FBS. NS/P cells were differentiated from Sox1-GFP knock-in ESC (46 C ESC) in neuronal culture medium (DMEM: neural basal medium = 1:1 supplemented with N2 and B27) as described previously^8, 9^. GFP positive neurospheres were used in all experiments. We used a previously described cell contact co-culture procedure^6^. Briefly, 3 × 10^5^ bEnd.3 cells were seeded onto 60 mm culture dishes for one day to allow attachment after which the medium was changed to neuronal culture medium. Sox1-GFP positive NS/P cells were then laid on top of the attached bEnd.3 cells to cover more than 80% of the attached bEnd cell surface and cultured for 24 hours. NS/P cells were then detached from bEnd.3 cells by repeated pipetting and bEnd.3 cells were then detached from culture dishes by trypsin^6^. In cultures where cholera toxin (CTX) or p38MAPK inhibitor SB20358, was tested, they were added directly to bEnd cells for 2 hours at 1ug/ml and 10 uM respectively before co-culture with NS/P cells. To detect phosphorylated β-catenin, proteasome inhibitor MG132 was added to NS/P-bEnd co-culture at 10 uM 2 hours before harvesting cells. Medium and reagents for cell culture were all purchased from Invitorgen. CTX and SB20358 were purchased from Sigma.

### MTP Variants preparation

The pcDNA3.1/myc-His (−) expression vector containing the full-length mouse MTP coding region carrying a point mutation within the MTP-siRNA recognition site^9^ was used as a template to prepare all the MTP variants which contain both myc and 6His tags at the C-terminus. The protease inactive form of MTP was prepared by changing Ser residue in the catalytic triad of the protease domain to Ala as described previously^9^. The primer sets used to generate the different MTP deletion variants were listed in Table 1. Resulting MTP protein constructs are shown in Fig. 2A. To delete the large fragment of MTP (Fig. 2A, variants 1, 2 and 3), the forward primer contains a BamHI sequence flanking the first ATG of MTP cDNA and the reverse primers contain XbaI sequences immediate upstream of the regions to be deleted. The resulting DNA fragments were then cloned into pcDNA3.1/myc-His(−) expression vector at the same restriction sites. Deletion of LDLR domains or CUB domains (Fig. 2A, variants 5 and 6) was made using the site-directed mutagenesis approach (Strategene) using complementary primer sets containing sequences flanking the regions to be deleted. After primer extension using PfuUltra High Fidelity DNA polymerase (Stratagene), the parental plasmid template was then digested with DpnI endonuclease, and the deletion-containing DNA was transformed into XL10-GoldUltra competent cells for nick repair and plasmid amplification. To make a construct containing the two CUB motifs, the same site-directed mutagenesis approach was performed using MTP variant 2 as template.

**Table 1:** MTP variants and the primer sets used to obtain them. ^a^

Variants: Description	Primer sets
1: 611-855 AA deletion	forward primer^b^5′-AGCGGATCCAAAACC**ATG**GGTAGCAA-3′
	reverse primer 5′-GCTTCTAGAGGTAAAGGATCGCAGCC-3′
2: 450-855 AA deletion	forward primer same as for variant 1
	reverse primer 5′-GCTTCTAGAGGAGTCGTAGGAGAGGT-3′
3: 210-855 AA deletion	forward primer same as for variant 1
	reverse primer 5′-GCGTCTAGAAGTCCTCTGCAGCATTC-3′
5: LDLR deletion	forward primer^c^
	5′-^1500^GAGTACTCTTCCTACGAC^1517^-^1989^CTGCGATCCTTTACCAAA^2006^-3′
	reverse primer
	complementary to the forward primer
6: CUB deletion	forward primer
	5′-^753^ACATCTGTGGTGGCCTTC^770^-^1518^TCCAACGACCCGTGCCCA^1535^-3′
	reverse primer
	complementary to the forward primer
CUB-motif construct	forward primer
	5′-^396^CTGGTGTGGCACTTCCAT^413^-^801^CAGGACAACAGCTGCAGT ^818^-3′
	reverse primer
	complementary to the forward primer

^a^The resulting protein constructs are shown in Fig. 2A and Fig. 3A.

^b^The underlined letters are the restriction enzyme sites included for cloning; the bold letters are the first methionine of MTP cDNA.

^c^3 or 4 digit shown in the primer sequences are the number of the nucleotides corresponding to the mouse MTP cDNA. The resulted MTP fragment will contain deletion between the two adjacent nucleotide numbered in the middle of the forward primer.

### Plasmid or siRNA Transfection

Transfection of MTP-expressing plasmid or siRNA was performed using Lipofectamin 2000 (for NS/P cells) or Lipofectamin 3000 (for bEnd.3 cells) as described previously^8, 9^. In brief, bEnd.3 cells were seeded on 60 mm^2^ culture dishes at 90% confluence then incubated with plasmid DNA (5 ug) or siRNA (10 nM) mixed with Lipofectamin 3000 (6 ul). For NS/P cell transfection, Lipofectamin 2000 was used. In cells that both expression plasmid and siRNA were transfected, cells were first incubated with siRNA-Lipofectamnin mixture for 48 hours, then incubated with expression plasmid for 24 hours. Overexpression or knockdown was checked by RT-PCR. Lipofectamin, the control siRNA with no target (non-silencing siRNA), specific siRNA against mouse St14 or MCAM were all purchased from Invitrogen.

### RNA isolation and RT-PCR

RNA isolation and RT-PCR were carried out as previously described^6, 8^. Gene-specific primers were as follows: mouse S15 (mouse ribosomal protein S15): forward 5′-TTCCGCAAGTTCACCTACC-3′, reverse 5′-TGCTTCACGGGTTTGTAGGT-3′; mouse MTP: forward 5′-CACTTCCATTATCGGAATGTGCG-3′, reverse 5′-GGATGTCGCCGGTCAGTATTGGTTATCA-3′; mouse MCAM: forward, 5′-GTGCGTCTTCTTGTTCGCTG-3′; reverse, 5′-ACATGGATGCCCACGACATT-3.

### Cross-linking of anti-His antibody to protein G magnetic sepharose beads

Protein G sepharose beads (purchased from GE Healthcare) 15 ul were incubated with 1 ug of antibody in TBS for two hours at room temperature. After brief equilibration (2 min) in 200 mM tri-ethanolamine (TEA) pH 8.9, cross-linking of antibody to the magnetic sepharose beads was achieved by one hour incubation at room temperature with 50 mM dimethyl dihydrochloride (DMP) in the same solution. DMP solution was removed, the beads were washed 2 min with 200 mM TEA pH 8.9 followed by 15 min wash in 100 mM ethanolamine.

### Isolation of MTP-interacting protein and LC-MS/MS spectrophotometer assay

Brain endothelial bEnd.3 cells transfected with expression vector carrying MTP CUB domains containing His and Myc tags were incubated with 20 mM dithiobissuccinimidyl propionate (DSP, Thermo) for 30 min at room temperature to covalently crosslink proteins that are in physical contact. DSP was removed; cells were incubated with 20 mM Tris-HCl pH 7.4 for 15 min at room temperature to stop the reaction. After washing twice with PBS, cells were collected by centrifugation and lysed in RIPA buffer. Five mg of cell lysate proteins were mixed with the antibody-crosslinked protein G magnetic sepharose beads and incubated overnight at 4 °C. Magnetic beads were collected by placing the tube on a magnetic rack and washed three times with RIPA buffer. The MTP-CUB domain associated proteins were released by incubation with 50 mM DTT in RIPA at 37 °C for 30 min. Collected protein solutions were pooled, trypsinized, and carbamidomethyl modified and sent to NHRI protein core facility for LC-MS/MS analysis. Peptide ion peaks from the LC/MS maps were detected with ProteinLynx Global Server (PLGS) software, MASCOT (www.matrixscience.com) was used to search MS/MS spectra for peptide/protein identification with SwissProt (UniProt) database. The MTP-CUB protein was released from the antibody-linked beads by 2 M urea in acid (pH 2.9) glycine-HCl solution (0.1 M).

### Immunoprecipitation (IP), Western blot, cell staining, and protein gel silver staining

Western blot and cell staining followed a procedure described previously^6, 8, 9^. Micro-Western screening was performed in the Protein Core facility of National Health Research Institutes (NHRI). Cell lysates of bEnd cells cultured alone, in contact co-culture with NS/P cells or in contact co-culture with MTP-knockdown NS/P cells were used in these screening. P38MAPK phosphorylation was used as internal control to monitor NS/P and bEnd cell direct contact^6^. Proteins exhibited the correct molecular size on micro-Western microgel, whose activation or inactivation occurred only in contact co-culture with NS/P cells and depended on the presence of MTP were selected. Molecules exhibiting all of these parameters in three independent preparation were picked for further examination by regular Western blot.

For the IP-Western, cells were lysed with RIPA buffer and clarified cell lysates were incubated overnight at 4 °C with indicated antibody. Protein G magnetic beads were then added and incubated for 3 hours. After washing with TBS, the bound proteins were released by boiling in SDS sample buffer and the released proteins were subjected to electrophoresis and Western blot analysis. Silver staining of protein gel was achieved using SilverXpress kit (Invitrogen) following the manufacturer’s procedure. Rabbit anti-GSK3β, anti-Ser9-phospho-GSK3β, anti-phospho-β-catenin antibodies and anti-p38MAPK were from Cell Signaling. Mouse anti-β-catenin antibody was from Millipore. Anti-6His antibody was from Genescript (A00174), anti-c-Myc was from Proteintech (60003-2-Ig), and anti-CD146 antibody was from R&D (MAB7718).

### Brain tissue preparation, sectioning and immunofluorescent staining

After fixed in 4% paraformaldehyde, mouse brains were washed and transferred to 30% sucrose. Tissues were left in sucrose solution at 4 °C until tissues no longer float. Brain tissues were embedded in O.C.T. in the coronal orientation and cut at 30 um with cryostat (Leica). Brain tissue sections were stained in floating condition. Tissue sections were blocked and permeabilized in PBS solution containing 10% horse serum and 0.5% Triton X-100 for 2 hours at room temperature. Following washing, tissues were incubated with primary antibody in PBS solution containing 4% horse serum and 0.1% Triton X-100 for overnight at 4 °C. Secondary antibody incubation was carried out in 4% horse serum/PBS. Nuclei were stained with Hoechst 33342, the tissue was mounted for image capture with Leica TCS SP6 II confocal microscope and the images were processed with LAS AF6000 system (NHRI optical core).

### Statistical analysis

Two-group t-test was used for comparison between groups. Data were presented as mean ± SEM derived from 3–5 independent experiments.

## Electronic supplementary material

supplementary
