# Supplementary material for: Physical Binding of Endothelial MCAM and Neural Transmembrane Protease Matriptase—Novel Cell Adhesion in Neural Stem cell Vascular Niche
Source: Sci Rep. 2017 Jul 10;7:4946. doi: 10.1038/s41598-017-05131-4 (PMC5504030; doi:10.1038/s41598-017-05131-4)
Supplement: Supplementary file 1 — supplementary [file 41598_2017_5131_MOESM1_ESM.pdf]

Supplementary materials for

Physical Binding of Endothelial MCAM and Neural Transmembrane Protease Matriptase—Novel Cell Adhesion in Neural Stem cell Vascular Niche

Hsiu-Hui Tung and Sheau-Ling Lee

Supplementary Figure 1. A. Original blot for variants 5 and 6 in Figure 2B. **B.** Original blot for variants 4 in Figure 2C. bEnd cells were cultured alone (Ctrl) or in contact co-cultured with MTP-knockdown NS/P cells (+siM NPC) the full-length wild-type MTP (pWT) or the indicated MTP variants (4, 5, or 6).

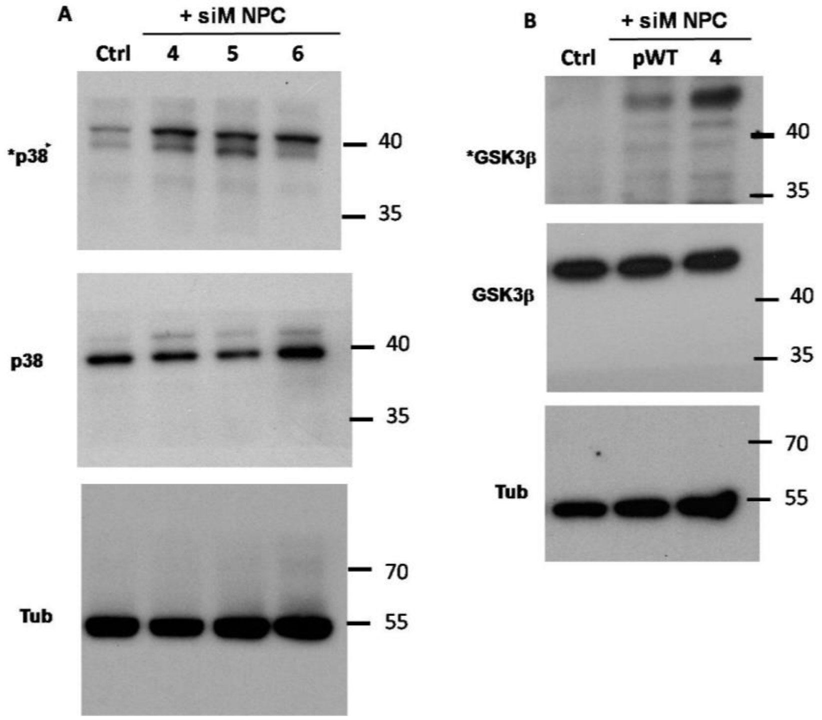

Supplementary Figure 2. Original blot for Figure 5A. Top panel shows Western blot with anti-MCAM antibody; bottom panel shows Western blot with anti-6His antibody for detection of MTP. Molecular sizes in kDa are indicated on the left of each blot. All the other detail refer to the Figure legend for Figure 5A.

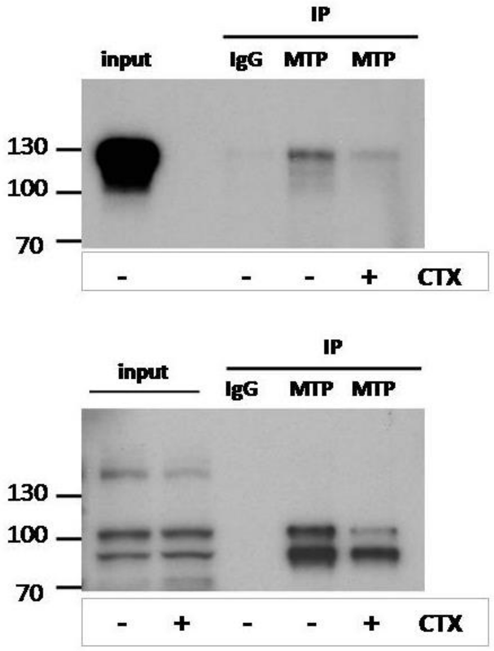

Table 1. LC-MS/MS summary of matched membrane molecules.

Matched protein	Peptide scores	Number of matched peptide
MCAM	336	7
Integrin β1	211	4
PECAM	188	9
F11r	91	2
